# Supplementary material for: VLP-ELISA for the Detection of IgG Antibodies against Spike, Envelope, and Membrane Antigens of SARS-CoV-2 in Indian Population
Source: Vaccines (Basel). 2023 Mar 27;11(4):743. doi: 10.3390/vaccines11040743 (PMC10145915; doi:10.3390/vaccines11040743)
Supplement: Supplementary file 1 [file vaccines-11-00743-s001.zip › vaccines-2254760-supplementary.pdf]

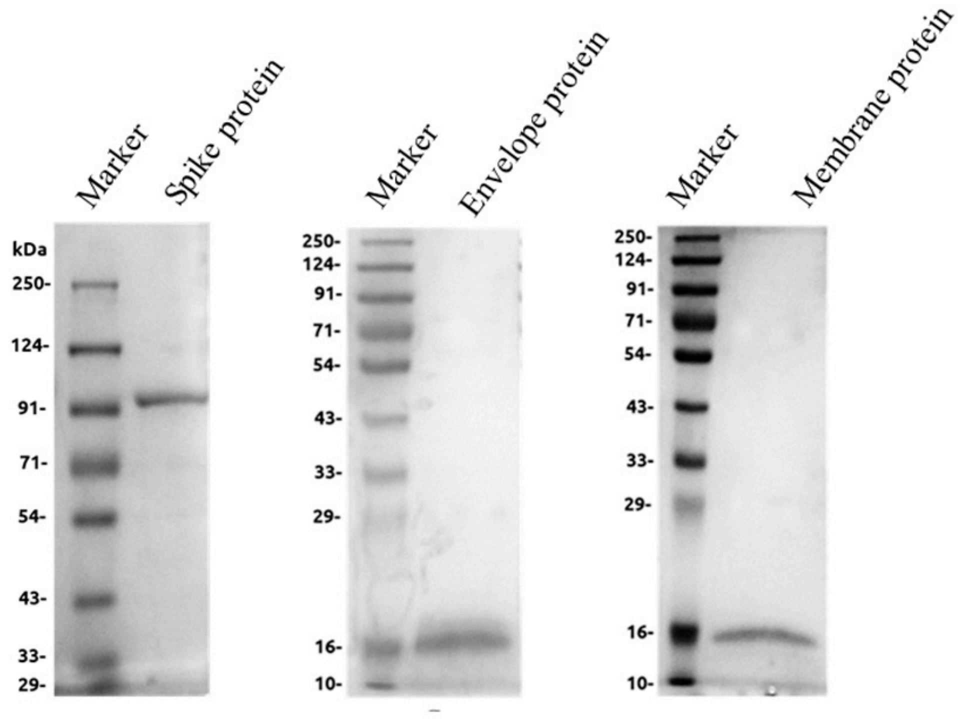

Figure S1. SDS-PAGE analysis of spike (S), envelope (E) and membrane (M) protein. ImageJ demonstrated that densitometric readings/intensity ratios for S, E, and M proteins were 1, 1.01, and 1, respectively.
